# Cognitive ageing among older Latin American immigrants: current evidence and limitations

**DOI:** 10.3389/fnagi.2026.1860593

**Published:** 2026-09-11

**Authors:** Fabiana Ribeiro, Teresa Capitao, Ana C. Teixeira-Santos

**Affiliations:** Department of Social Sciences, Institute for Research on Socio-Economic Inequality, University of Luxembourg, Esch-sur-Alzette, Luxembourg

**Keywords:** ageing, cognition, immigrants, inequalities, Latin America

## Abstract

**Objective:**

This review synthesises current evidence on cognitive function among older Latin American immigrants and identifies key factors associated with cognitive outcomes.

**Methods:**

Following PRISMA guidelines, a systematic search was conducted across five databases (Embase, PubMed, Web of Science, Scielo, and Scopus) for peer-reviewed quantitative studies, without language restrictions. Titles and abstracts of 188 records were screened, and nine articles were selected for full-text screening.

**Results:**

Overall, three studies met the inclusion criteria, all conducted in the United States of America and primarily involving Mexican-born older adults, revealing the limited geographic and ethnic diversity of the available evidence. Cognitive outcomes were assessed using standardised global cognition measures and compared with those of non-immigrant populations. These disparities were largely associated with socioeconomic disadvantages and lower educational achievements. Additional factors consistently linked to cognitive outcomes included gender, cardiometabolic health, country of origin, age at migration, and neighbourhood socioeconomic context.

**Conclusion:**

Evidence on cognitive ageing among older Latin American immigrants remains limited and geographically concentrated. Greater research diversity and more precise characterisation of immigrant populations are needed to better understand cognitive ageing and to inform equitable, culturally responsive policies and interventions.

## 1 Introduction

Population ageing and international migration are two major demographic trends restructuring global health landscapes. In recent decades, migration from Latin America to high-income countries has increased substantially, especially into North America (United Nations, 2024). As this population ages, understanding determinants of cognitive health becomes crucial, given the rising burden of cognitive impairment and dementia worldwide (Liu et al., 2025).

It is important to note that cognitive ageing is shaped by a complex interplay of factors, including early-life socioeconomic conditions, education, health behaviours, access to healthcare and gender/sex (Livingston et al., 2020, 2024; Ribeiro et al., 2021; Ribeiro et al., 2022; Rodrigues et al., 2025; Ribeiro et al., 2023). Among immigrant populations, cognitive ageing is further influenced by migration-related factors, including acculturation, age at migration, and exposure to structural inequalities in host societies (Qi et al., 2025; Loi et al., 2026). Additionally, migration-related selection may also play a role, as migrants may differ systematically from those who remain in their country of origin, reflecting a potential selection mechanism known as the “healthy migrant effect” (Ruiz et al., 2013). These factors could either exacerbate or mitigate the risk of cognitive decline.

Several studies have explored the Hispanic paradox, a phenomenon mainly described in the U.S. literature, whereby Hispanic/Latino populations experience more favourable health outcomes than what would be expected based on their socioeconomic profiles, including lower mortality and morbidity, despite higher levels of social disadvantage (Markides and Coreil, 1986; Ruiz et al., 2013). Although the Hispanic paradox has often been discussed in relation to migration-related explanations, such as the healthy migrant effect, longitudinal evidence indicates that the Hispanic mortality advantage is also observed in older populations. A systematic review and meta-analysis found an overall 17.5% lower mortality risk among Hispanic populations and showed that this advantage became stronger with increasing participant age (Ruiz et al., 2013).

Whether this late-life mortality advantage extends to cognitive ageing remains unclear. Evidence on cognitive outcomes suggests a more complex pattern. In models adjusted only for age and gender, foreign-born Mexican Americans appeared to be at higher risk of cognitive impairment (Weden et al., 2017). However, after adjustment for individual and neighborhood socioeconomic factors, this association was reversed, revealing lower odds of prevalent cognitive impairment and lower hazards of incident cognitive impairment among foreign-born Mexican Americans. These results suggest that the potential cognitive advantages associated with the Hispanic paradox may be masked by structural inequalities. Cognitive ageing disparities across racial and ethnic groups are strongly shaped by socioeconomic disadvantage, particularly differences in education, income, and neighbourhood conditions (Haan et al., 2011; Matyi et al., 2025). Furthermore, existing studies highlight potential mechanisms underlying cognitive disparities, including educational inequalities, cardiometabolic risk, and cumulative exposure to socioeconomic disadvantage across the life course (Glymour and Manly, 2008; Haan et al., 2011; Matyi et al., 2025). Additionally, contextual factors such as neighbourhood socioeconomic conditions and residence in immigrant regions may influence health outcomes through mechanisms including social support, stress buffering, and access to resources (Diez Roux, 2001). Overall, the findings highlight the complex interplay among socioeconomic disadvantage, migration-related selection, and contextual factors in shaping cognitive ageing among Latin American immigrants.

Despite growing interest in inequalities among immigrant populations, the literature is limited, with few studies focusing specifically on cognition in older Latin American immigrants. This limited evidence may reflect the difficulty of identifying studies that clearly distinguish older first-generation Latin American immigrants from broader ethnic categories such as “Hispanic” or “Latino.” It may also reflect broader barriers to research participation and representation, including systemic racism, medical and institutional mistrust, concerns related to safety and immigration or citizenship status, language barriers, and limited opportunities for research to be conducted, led, and informed by Hispanic/Latino communities (Aranda et al., 2023; Rodriguez et al., 2023). Given these limitations, this review aims to systematically examine studies investigating inequality in cognitive function among older Latin American immigrants compared to their native-born peers through quantitative cognitive measures. More specifically, we aim to clarify what has already been investigated, identify patterns in cognitive functioning among older Latin American immigrants, and highlight methodological limitations and research gaps.

## 2 Method

### 2.1 Search strategy

This review was conducted in accordance with the Preferred Reporting Items for Systematic Reviews and Meta-Analyses (PRISMA) guidelines (Page et al., 2021; Supplementary material S1). A search strategy was developed to identify peer-reviewed observational studies (see Supplementary materials S2, S3). Systematic searches were conducted in Embase, PubMed, Web of Science, Scielo, and Scopus on 30 December 2025. No restrictions on language or publication date were applied. The review protocol was registered in PROSPERO (CRD420261299157).

### 2.2 Criteria for inclusion and exclusion of studies

All records retrieved from the databases were imported into Microsoft Excel, and duplicates were removed. Two reviewers independently screened titles and abstracts and subsequently assessed the full texts of potentially eligible studies against the prespecified eligibility criteria. Studies were eligible if they included Latin American first-generation immigrants aged ≥50 years, who, at the time of data collection, were residing outside their country of birth and whose immigrant status could be clearly determined. Eligible studies also had to compare these participants with native-born individuals in the host country. Studies were required to include only participants aged ≥50 years or to report data separately for this age group; provide sufficient information to determine that participants were Latin America immigrants born outside the host country and residing in the host country at the time of data collection; and assess cognitive functioning through a clinician-established diagnosis, a validated brief cognitive instrument (e.g., the Mini-Mental State Examination), or domain-specific neuropsychological tests.

We excluded non-original publications, studies including younger participants without separately reported data for individuals aged ≥50 years, studies without a clearly defined native-born host-country comparison group, including studies comparing first-generation Latin American immigrants only with second generation, studies in which current immigrant status could not be established (e.g., studies of return migrants who were residing in their country of birth at the time of data collection), studies assessing only subjective cognitive decline, and studies using broad ethnic labels such as “Hispanic” without specifying Latin American country of origin, country of birth, or immigration status. Disagreements between reviewers were resolved through discussion and consensus.

### 2.3 Data extraction and quality assessment

Data were extracted using a form that included study characteristics, population demographics, migration-related variables, cognitive outcomes, and key findings. One reviewer performed data extraction and independently checked by a second reviewer. Furthermore, a narrative synthesis approach was used to summarise findings across studies, focusing on similarities and differences in study design, populations, cognitive outcomes, and associated factors.

Risk of bias and methodological quality were assessed using the Newcastle–Ottawa Scale for longitudinal studies and the Joanna Briggs Institute Critical Appraisal Checklist for analytical cross-sectional studies. Discrepancies were resolved by consensus.

## 3 Study selection

**Figure 1:**
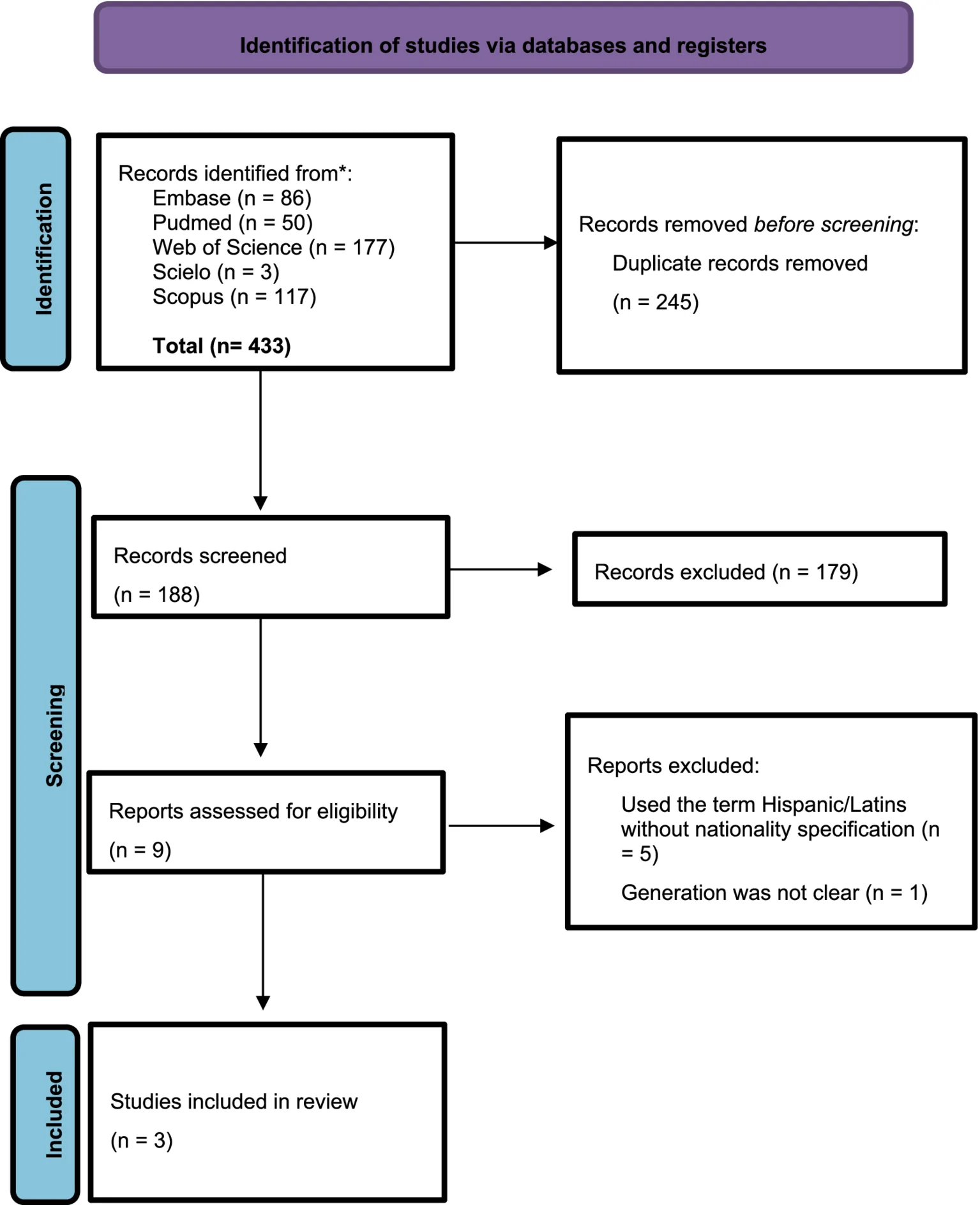
Flow diagram of the study selection process.

The search identified 433 potentially relevant records. After removing duplicates, 188 articles were assessed for eligibility and titles and abstracts were screened. Of these, 9 papers were selected for full-text review, and 3 studies met the inclusion criteria and were included in the final review. Consequently, six articles were excluded, with the reasons for exclusion detailed in Figure 1.

## 4 Results

### 4.1 Characteristics of the selected studies

All three studies were conducted in the United States of America and published in English. Two of the studies used data from the Health and Retirement Study (Casanova and Aguila, 2020; Kezios et al., 2023), while one used both the Health and Retirement Study and the Mexican Health and Ageing Study (Casanova and Aguila, 2020). Two studies compared Mexican-origin groups (foreign- vs. U.S.-born) with non-Hispanic Whites (Casanova and Aguila, 2020; Kezios et al., 2023), while Weden et al. (2017) compared foreign-born Mexican Americans, U.S.-born Mexican Americans, and U.S.-born non-Hispanic Whites and Blacks. Sample sizes ranged from 994 foreign-born Mexican Americans to over 70,000 non-Hispanic Whites. One study stratified analyses by sex (Casanova and Aguila, 2020), whereas others pooled samples with demographic adjustments (Weden et al., 2017; Kezios et al., 2023). Only one study was cross-sectional (Casanova and Aguila, 2020), while (Kezios et al., 2023) evaluated cognitive trajectories over a 24-year follow-up (1994–2018), whereas Weden et al. (2017) assessed the incidence of cognitive impairment during a 10-year follow-up (2000–2010). Age was handled differently across studies. Casanova and Aguila (2020) included participants aged 50–80 years and observed that Mexican immigrants were the youngest group, whereas return migrants were the oldest. Kezios et al. (2023) treated age as a continuous variable centred at 70 years in the statistical models. Weden et al. (2017) presented mean ages in the early 60s and adjusted for age in all multivariable analyses (Table 1).

**Table 1:** Characteristics of the included studies.

Author and publication year	Country (Region)	Sample Size	Sampling Study	Age (range)	Gender (%)	Cognitive tests used	Primary outcome
Casanova and Aguila (2020)	USA	Core analysis: 78,248; non-Hispanic whites (*n* = 74,882) and Mexican immigrants (*n* = 3.366). HRS-MHAS selection analysis: 28,322 person-wave observations	HRS - RAND version, version P; MHAS	50–80	HRS core analysis: ~42% male and ~58% female (varies by analysis) HRS-MHAS analysis: ~ 43% male and ~57% female	Total Word Recall (Immediate and Delayed Word Recall), Serial 7 s, Date Naming, Backwards Counting, Object Naming	Total word recall, Serial 7 s, and date naming were treated as continuous outcomes using gender-specific standardised z-scores and analysed with OLS regressions. Backward counting and object naming were treated as binary outcomes and analysed with Probit models. Analyses were conducted separately by gender, with sequential adjustment for demographics, education, income, health conditions, weight, smoking, and depressive symptoms.
Kezios et al. (2023)	USA	22,584 foreign-born Mexican American living in the USA (*n* = 994), US-born Mexican Americans (*n* = 968) and US-born non-Hispanic whites (*n* = 20,622)	HRS	≥50	~47% males ~53% females	Harmonised global measure of cognition (continuous z-score) developed under the PITCH project using item response theory	Global cognition was treated as a continuous z-score. The authors used linear mixed models with age as the time scale to estimate baseline differences and cognitive change over time. Models were unadjusted and adjusted using conventional regression and propensity score-based approaches, including inverse probability weighting, trimming, and match weighting.
Weden et al. (2017)	USA	8,433 for prevalent cognitive impairment 7,076 cognitively normal participants for incident cognitive impairment analyses	HRS	≥51	47% males 53% females	Modified 27-point English and Spanish Telephone Interview for Cognitive Status, proxy- and interviewer-assessments of cognition, IADL	Prevalent and incident cognitive impairment - respondents with a score of 11 or less were classified as having Cognitive impairment TICS; including immediate and delayed word recall, Serial 7 s, and backward counting; proxy- and interviewer-based assessments of cognition and IADLs for proxy respondents

For Casanova and Aguila (2020), values refer to person-wave observations rather than unique individuals; the HRS core analysis included repeated observations across waves 5–11, whereas the combined HRS-MHAS analysis was used to examine migrant selection and included Mexican immigrants in the HRS, return migrants in the MHAS, and non-migrants in the MHAS. HRS, health and retirement study; IADL, instrumental activities of daily living; MHAS, Mexican Health and Aging Study; OLS, ordinary least squares; PITCH, psychometric integrative technology for cognitive health; PS, propensity score; RAND-HRS, RAND version of the health and retirement study; TICS, telephone interview for cognitive status; U.S., United States of America.

### 4.2 Measures of cognitive functioning

All studies assessed cognition using standardised survey-based measures rather than clinical evaluations, although the assessment approaches differed. Casanova and Aguila (2020) evaluated five cognitive domains through individual tasks assessing episodic memory (total word recall), attention/working memory, processing speed (Serial 7 s/ backward counting), temporal orientation (date naming), and language (object naming). Kezios et al. (2023) used a harmonised global cognition z-score derived from the Health and Retirement Study (HRS) across waves from 1994 to 2018. Instead of analysing individual cognitive tasks, the authors employed a continuous global cognition measure developed through the Psychometric Integrative Technology for Cognitive Health (PITCH) project, which used item response theory to harmonise cognitive assessments across the HRS. Weden et al. (2017) defined cognitive impairment using a validated 27-point modified version of the Telephone Interview for Cognitive Status (TICS), available in both English and Spanish, which incorporates immediate and delayed word recall, Serial 7 s, and backward counting.

### 4.3 Covariates included in the statistical analyses

Covariates varied across studies but generally included demographic and socioeconomic characteristics, such as age, sex, education, marital status, and income or wealth. Casanova and Aguila (2020) additionally adjusted for health-related factors, including hypertension, diabetes, heart disease, stroke, body mass index, smoking, and depressive symptoms. Migration-related variables were also considered, although their operationalisation differed between studies. Casanova and Aguila (2020) examined age at migration, whereas Kezios et al. (2023) included migration selection factors such as parental education, age at first job, age at first marriage, height, and smoking initiation, with additional sensitivity analyses incorporating childhood socioeconomic conditions. Weden et al. (2017) further accounted for contextual characteristics, including neighbourhood socioeconomic status and immigrant enclave measures.

### 4.4 Main findings

Overall, Casanova and Aguila (2020) found that Mexican immigrants had lower cognitive performance than non-Hispanic Whites across all cognitive measures. These differences were observed in models adjusted for demographic characteristics, including age, marital status, and survey waves. The authors then estimated a sequence of progressively adjusted models. First, they added education, followed by household income, to assess the role of socioeconomic factors. Subsequent models further adjusted for potential dementia-related risk factors, including cardiovascular conditions, body weight, smoking behavior, and depressive symptoms. Socioeconomic factors, particularly education and income, explained a substantial part of the cognitive gap. Among men, the differences between Mexican immigrants and non-Hispanic Whites became statistically non-significant after adjustment for education and income. Among women, however, cognitive scores remained significantly lower among Mexican immigrants even after socioeconomic adjustment, indicating a persistent gender-specific disadvantage. Additional adjustment for health conditions, body weight, smoking, and depressive symptoms produced only minor changes in the estimates.

Similarly, Weden et al. (2017) reported that both U.S.-born and foreign-born Mexican Americans initially showed a higher risk of cognitive impairment than U.S.-born non-Hispanic Whites in models adjusted only for age and gender. These differences were substantially attenuated after adjustment for individual and neighbourhood socioeconomic factors, including education, wealth, and neighbourhood socioeconomic status. Notably, among foreign-born Mexican Americans, the association was reversed after socioeconomic adjustment, suggesting a potential immigrant advantage in cognitive impairment once socioeconomic disadvantage was taken into account.

In contrast, Kezios et al. (2023) found that both foreign-born and U.S.-born Mexican Americans had lower baseline global cognition scores than U.S. born non-Hispanic Whites. However, their rates of cognitive decline were like, or slower than, those observed in the U.S.-born non-Hispanic Whites. These findings remained consistent after adjustment for migration selection factors and after applying propensity score methods to improve covariate overlap between groups. Overall, the study suggested that although Mexican Americans exhibited lower baseline cognitive performance, they did not experience faster cognitive decline over time.

### 4.5 Quality assessment

The two longitudinal studies (Kezios et al., 2023; Weden et al., 2017) were assessed using the Newcastle–Ottawa Scale and were judged to be of high methodological quality. Both studies demonstrated important strengths, including representative cohorts, validated cognitive measures, adjustment for key confounders, and sufficiently long follow-up periods. Kezios et al. (2023) received 8/9 stars, with one star not awarded because participants of Mexican background entered the HRS at later waves, reducing their repeated cognitive assessments compared with U.S.-born non-Hispanic White participants (see Supplementary material S4).

## 5 Discussion

The main goal of this review was to systematise existing studies and explore cognitive ageing inequalities affecting Latin American immigrants in comparison with non-immigrant populations, while also identifying the social determinants that may contribute to or explain these disparities. Despite the topic’s relevance, the scientific literature was extremely limited. In this context, only three studies met the inclusion criteria: all were conducted in the United States, focused on Mexican immigrants, and were based mainly on the HRS, which is a longitudinal cohort study of older adults in the United States of America designed to examine ageing, health, retirement, and socioeconomic dynamics over time, initiated in 1992 (Sonnega et al., 2014). The included studies did not represent Latin American immigrants as a broad and diverse population. Instead, the evidence was concentrated almost exclusively on Mexican-origin populations in the United States. As a result, the findings may not be generalisable to Latin American immigrants from other countries of origin, who may differ in migration trajectories, socioeconomic conditions, legal status, racialisation processes, and host-country contexts.

Studies included in this review have consistently shown that Mexican-origin populations exhibit poorer baseline cognitive performance compared with native-born populations. These results are in concordance with a previous systematic review showing a higher prevalence of cognitive decline in Latin American countries compared to highly developed countries (Ribeiro et al., 2022). However, this difference appears to be largely explained by socio-economic inequalities, particularly in terms of educational attainment, underscoring the importance of life-course conditions for cognitive ageing (Glymour and Manly, 2008; Haan et al., 2011; Livingston et al., 2024). These findings are consistent with the literature highlighting the role of the accumulation of social disadvantages from childhood onwards in determining cognitive health in old age (Geraets et al., 2026). However, the evidence suggests that this mechanism alone is not sufficient. Factors such as acculturation, age at the time of migration, and exposure to inequalities in the destination country also appear to play a significant role in shaping cognitive trajectories (Qi et al., 2025; Loi et al., 2026), although these have yet to be fully explored empirically.

Furthermore, the findings point to a more complex pattern. Studies among Mexican-origin populations in the United States of America suggest that, after adjusting for socio-economic disadvantage and migration-related selection factors, foreign-born Mexican Americans may show lower risk of cognitive impairment or similar/slower rates of cognitive decline compared with U.S.-born comparison groups (Weden et al., 2017; Kezios et al., 2023). One of the most widely accepted explanations is that of ‘migratory selection’ or ‘Hispanic paradox’, according to which healthier or more resilient individuals are more likely to migrate, which may contribute to the observed results (Ruiz et al., 2013). In the case of cognition, it proves to be partial and dependent on controlling for structural factors, such as reduced access to lifelong education, more precarious employment, greater exposure to risks, barriers to healthcare access and/or living in neighbourhoods with fewer resources. Rather than a clear advantage, the findings suggest the coexistence of potential protective factors and persistent social disadvantage (Diez Roux, 2001; Weden et al., 2017).

Another factor that has received little attention in the field concerns gender inequalities. Just one study carried out gender-stratified analyses, showing that cognitive disparities differed between Mexican immigrant men and women (Casanova and Aguila, 2020). Their findings suggest that immigrant women may experience poorer cognitive outcomes even after adjusting for socioeconomic factors, whereas cognitive differences among immigrant men appeared to be largely explained by these factors. This may reflect inequalities accumulated over a lifetime, including educational opportunities, reasons for migration, labour market participation, and caregiving responsibilities, which negatively impact cognitive health in old age (Ribeiro et al., 2023).

Moreover, despite using a common data source, variation in cognitive measurement approaches might also have contributed to differences in findings. Studies differed in whether they examined specific cognitive domains, global cognitive scores, or categorical cognitive impairment, and in whether they focused on cross-sectional differences or longitudinal trajectories. These methodological differences limit direct comparability. Furthermore, none of the studies used clinical diagnostic criteria for dementia or mild cognitive impairment. Instead, they relied on survey-based cognitive assessments, which, although validated and useful for population-based research, may not fully capture clinically diagnosed cognitive disorders or subtle cognitive changes.

The quality assessment indicated that the overall evidence base was of moderate-to-high methodological quality, with strengths including large population-based samples, standardised cognitive measures, and adjustment for key demographic and socioeconomic factors. The longitudinal studies by Kezios et al. (2023) and Weden et al. (2017) provided stronger evidence because they assessed cognitive change or incident cognitive impairment over time. Nevertheless, residual confounding may remain, particularly in relation to migration history, early-life socioeconomic circumstances, and other factors associated with migrant selection.

The cross-sectional study by Casanova and Aguila (2020), although methodologically robust, provided only a single assessment of cognitive function and therefore cannot assess trajectories of cognitive decline or clarify how life-course processes, including the accumulation of socioeconomic disadvantage, contribute to observed differences between immigrant and non-immigrant populations (Glymour and Manly, 2008; Haan et al., 2011). However, potential selection bias is not limited to cross-sectional designs and may affect population-based studies more broadly. Older Latin American immigrants who participate in such studies may differ systematically from those who do not. Factors such as migration-related health selection, language barriers, differential survey participation, loss to follow-up, and return migration may influence the interpretation of observed cognitive differences.

Taken together, the findings suggest that the inequality in cognitive ageing among Latin American immigrants results from a complex interplay between life-course factors, as well as sociodemographic and contextual characteristics. Moreover, the available literature has significant limitations. The concentration of studies in the United States of America and their predominant focus on populations of Mexican origin limits the generalisability of the findings to other national contexts and Latin American immigrant groups. This concentration is partly understandable, given that people of Mexican origin constitute the largest Latin American-origin population in the United States of America, thereby providing larger and more feasible samples for population-based research. Conversely, research involving smaller Latin American-origin groups may be constrained by limited sample sizes, fewer dedicated research resources, and structural, linguistic, and recruitment barriers affecting participation. Migration histories and policies, healthcare systems, social conditions, and patterns of social integration may also differ considerably across receiving countries and Latin American communities, reinforcing the need for research involving more diverse populations and settings.

Additionally, several studies identified in the literature relied on broad ethnic categories such as “Hispanics” or “Latinos.” These labels, which have long been criticised for their conceptual ambiguity (Gimenez, 1989). In particular, they may combine foreign-born immigrants, second-generation individuals, and long-established populations, without distinguishing specific country of origin or nativity. For this reason, we excluded studies in which participants’ migration status could not be clearly determined.

It is important to acknowledge that we used rigorous inclusion criteria to ensure that the synthesised evidence was directly applicable to older, first-generation Latin American immigrants and that between-group differences could be interpreted in relation to immigrant status. Requiring explicit information on age, country of birth, country of origin, and current immigrant status reduced population misclassification and heterogeneity across age groups, immigrant generations, and populations with distinct sociocultural and migration experiences. Although these criteria limited the number of eligible studies, they strengthened the specificity, methodological consistency, and interpretability of the synthesis.

Future studies should prioritise more diverse samples that include a wider range of Latin American groups and, where possible, incorporate subgroup analyses to capture within-group heterogeneity that is often obscured by aggregated categories. Additionally, future research should adopt more detailed measures of the migration experience, such as age at migration, length of residence and migration trajectories, to capture the multidimensional nature of migration and its potential implications for cognitive ageing. Other relevant determinants, such as acculturation, bilingualism, educational quality and literacy, discrimination, social support, and depression, were not examined consistently in the included studies and should be prioritised in future research. The inclusion of life-course approaches and intersectional perspectives, including gender, is also essential to better understand how different forms of inequality accumulate and interact over time. Ultimately, this study reinforces the need to understand cognitive disparities between Latin American immigrants and non-immigrants, a population that remains both underrepresented in cognitive ageing research and exposed to social determinants that may shape cognitive trajectories. Addressing these gaps is essential not only to advance scientific knowledge but also to identify the mediating mechanisms that drive these differences and to inform policies aimed at reducing inequities across ageing populations.
